# Pushing the boundaries of total scattering methods

**DOI:** 10.1107/S2052252519002847

**Published:** 2019-03-01

**Authors:** Robert J. Koch

**Affiliations:** a Condensed Matter Physics and Materials Science, Brookhaven National Laboratory, Upton, NY 11973, USA

**Keywords:** commentary, grazing incidence, pair distribution function, thin films

## Abstract

Atomic pair distribution (PDF) analysis has proven to be an exceptionally robust tool for probing the structure of amorphous, crystalline and crystallographically challenged materials. This issue of **IUCrJ** features a significant step forward in X-ray PDF methodology for thin films, with substantial improvements in both sensitivity and time resolution.

Total scattering and atomic pair distribution function (PDF) analysis, an extension of powder diffraction, has proven exceptionally useful for probing both local and long-range structure across a wide variety of materials (Billinge & Kanatzidis, 2004 ▸; Egami & Billinge, 2012 ▸; Keen & Goodwin, 2015 ▸). The application of PDF analysis was initially limited to materials completely lacking long-range order (Guinier, 1994 ▸; Warren, 1969 ▸). Over the last two decades, developments in instrumentation (Chupas *et al.*, 2003 ▸) and software (Proffen & Billinge, 1999 ▸; Proffen & Neder, 1999 ▸; Peterson *et al.*, 2000 ▸; Billinge *et al.*, 2002 ▸; Qiu *et al.*, 2004 ▸; Tucker *et al.*, 2007 ▸; Juhás *et al.*, 2013 ▸) have allowed scientists to leverage computational advances and bring PDF to the forefront of structural characterization, extending its applicability well beyond amorphous materials. In this issue of **IUCrJ**, Dippel *et al.* (2019 ▸) report a new method for carrying out grazing-incidence PDF (GIPDF) measurements. This optimized GIPDF configuration has the potential to broaden the horizons of the total scattering field even further.

The PDF *G*(*r*) is obtained through a through a Fourier transform of the normalized diffraction intensity *I*(*Q*) measured up to a momentum transfer *Q* typically greater than 20 Å^−1^. While *G*(*r*) and *I*(*Q*) contain identical information, the PDF provides a unique ‘real-space’ view, giving the likelihood of finding pairs of atoms at a certain distance *r*. As assumptions regarding periodicity are not necessary, PDF analysis has proven particularly useful for nanomaterials (Masadeh *et al.*, 2007 ▸; Page *et al.*, 2011 ▸), materials with poor or only anisotropic crystallinity (Vogt *et al.*, 2002 ▸; Gao *et al.*, 2017 ▸) and crystalline systems with local structural distortions (Billinge *et al.*, 1996 ▸; Senn *et al.*, 2016 ▸).

Total scattering techniques place Bragg and diffuse scattering on equal footing, and thus it is ideal if the signal represents only the material of interest. This necessitates careful measurement and subtraction of the scattering signal from non-sample (background) materials in the beam path. For bulk powders measured in the standard transmission geometry at modern synchrotrons, this is usually straightforward, as the signal-to-background ratio is large.

In the case of thin films on thick substrates, background subtraction in a transmission geometry is critically important and at times a limitation. As film thicknesses decreases relative to the substrate, the contribution of the film to the scattered signal similarly decreases. The result is often that the measured signal is dominated by the substrate, while the signal from the film of interest is buried in noise. While this issue can be remedied by increasing measurement times compared with those for standard bulk powders, the necessary times are often unfeasible. This has limited the applicability of PDF analysis to thin films, imposing hard restrictions on both film thickness and temporal resolution.

The recent work of Dippel *et al.* (2019 ▸) represents a significant advancement in the field of X-ray total scattering and PDF analysis. They exploit the grazing-incidence geometry shown inset in Fig. 1 ▸, where the X-ray beam meets the sample at an angle below that of total external reflection. In such a configuration, under ideal conditions the penetration depth of X-rays is independent of wavelength, and typically less than a nanometre, as shown in Fig. 1 ▸. This provides enhanced surface sensitivity and a significantly improved signal-to-background ratio, driving down the thickness detection limit.

Their work makes several key improvements on past GIPDF geometries. By using hard X-rays combined with a fast two-dimensional detector they achieve PDF resolutions and count times comparable with what is seen in more standard transmission geometries for bulk powders. Additionally, careful control over the beam footprint yields manageable instrumental resolution broadening. Using beamline P07 at PETRA III, Dippel *et al.* successfully measured PDFs from crystalline and amorphous thin films down to 3 nm, with count times on the order of one second. This represents a factor of ten decrease in the minimum detectable film thickness, and a decrease of several orders of magnitude in the measurement time. Beyond this, their subsequent analysis results agree with previous studies on the same materials, demonstrating robustness and reproducibility.

Thin films are particularly relevant in technological applications including energy storage and conversion (Gao *et al.*, 2017 ▸; Yang *et al.*, 2017 ▸; Mao *et al.*, 2017 ▸), catalysis (Morales-Guio *et al.*, 2016 ▸; Ng *et al.*, 2016 ▸; Xu *et al.*, 2016 ▸), and electronics (Martin & Rappe, 2017 ▸; Kelly *et al.*, 2017 ▸; Deng *et al.*, 2017 ▸). Dippel *et al.* have successfully demonstrated the feasibility of the GIPDF technique, bringing within reach a whole new class of *in situ* and *operando* structure studies on thin film devices and functional materials.

## Figures and Tables

**Figure 1:**
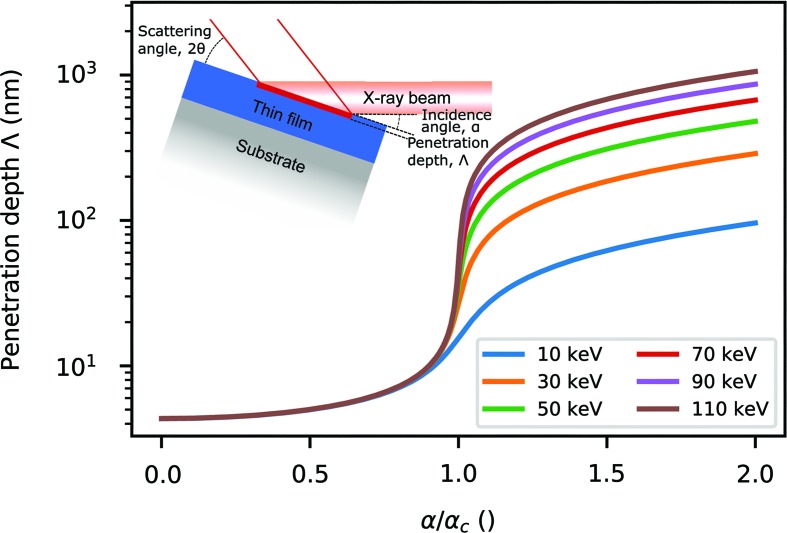
The penetration depth Λ of X-rays in copper metal as a function of incidence angle α relative to the critical angle for total external reflection, α_c_. Various energies are shown. For the grazing-incidence geometry (inset for reference) the penetration depth is small and energy independent for α < α_c_. This allows for selective enhancement of the thin film total scattering signal. Calculated based on equation (8) in the work of Feidenhans’l (1989 ▸).
